# eHealth Literacy, Online Help-Seeking Behavior, and Willingness to Participate in mHealth Chronic Disease Research Among African Americans, Florida, 2014–2015

**DOI:** 10.5888/pcd13.160210

**Published:** 2016-11-17

**Authors:** Delores C.S. James, Cedric Harville

**Affiliations:** Author Affiliation: Cedric Harville II, Department of Health Education and Behavior, University of Florida, Gainesville, Florida.

## Abstract

**Introduction:**

The high rate of ownership of smartphones among African Americans provides researchers with opportunities to use digital technologies to reduce the prevalence of chronic diseases in this population. This study aimed to assess the association between eHealth literacy (EHL) and access to technology, health information–seeking behavior, and willingness to participate in mHealth (mobile health) research among African Americans.

**Methods:**

A self-administered questionnaire was completed by 881 African American adults from April 2014 to January 2015 in north central Florida. EHL was assessed by using the eHealth Literacy Scale (eHEALS) with higher scores (range, 8–40) indicating greater perceived skills at using online health information to help solve health problems.

**Results:**

Overall eHEALS scores ranged from 8 to 40, with a mean of 30.4 (standard deviation, 7.8). The highest score was for the item “I know how to find helpful health resources on the Internet,” and the lowest score was for “I can tell high quality from low quality health resources on the Internet.” Most respondents owned smartphones (71%) and searched online for health information (60%). Most were also willing to participate in health research that used text messages (67%), smartwatches/health tracking devices (62%), and health apps (57%). We found significantly higher eHEALS scores among women, smartphone owners, those who use the Internet to seek health information, and those willing to participate in mHealth research (*P* < .01 for all).

**Conclusion:**

Most participants owned smartphones, used the Internet as a source of information, and were willing to participate in mHealth research. Opportunities exist for improving EHL and conducting mHealth research among African Americans to reduce the prevalence of chronic diseases.

## Introduction

Searching online for health information is an easy and affordable way for Americans to learn more about their health, self-diagnose an illness, and manage a health condition (1). Approximately 6 in 10 American adults search online for health information, and the trend is expected to increase as ownership of mobile devices grows and access to high-speed Internet expands (1). Knowing how to access and use credible online health information will allow patients to be more informed in medical decision making. This may ultimately impact health care costs, health outcomes, health care quality, and health equity (2).

Health literacy is a major public health concern and is one of 20 key areas identified to improve health outcomes and health care quality (2,3). African Americans have a disproportionately high prevalence of chronic diseases compared with other populations, and it is in the nation’s best interest to explore how new and emerging technologies can help to reduce these health disparities (2,4,5). One proposed way to reduce health disparities is to close the gap in health literacy and increase the use of health information technology to support patient self-management (2,6). Achievement of these objectives could also have an impact on eHealth literacy (EHL). EHL is “the ability to seek, find, understand, and appraise health information from electronic sources and apply the knowledge gained to addressing or solving a health problem” (7). The creation of an eHealth-literate population in an age of rapidly advancing technology should be a priority in American public health policy, research, practice, and education.

Despite persistent health disparities and the National Institutes of Health Revitalization Act of 1993, which mandated the inclusion of racial/ethnic minorities in all federally funded research (8), African Americans are underrepresented in eHealth research, clinical interventions, and clinical trials (2,9–11). Factors contributing to the underrepresentation of African Americans in research include institutional racism and historical mistrust of the health care system, research, and the government because of previous unethical research practices (12–15). Understanding EHL among African Americans and recruiting them into mobile health (mHealth) interventions are important goals for several reasons. African Americans 1) have a high prevalence of chronic diseases, 2) are the fastest adopters of home broadband Internet compared with other racial/ethnic groups, 3) have one of the highest rates of ownership of smartphones among racial/ethnic groups, and 4) have reported a willingness to participate in mHealth research (2,16–20). The objective of this study was to assess and examine the association between EHL and access to technology, health information–seeking behavior, and willingness to participate in mHealth research among African Americans.

## Methods

A self-administered questionnaire was completed by 903 African Americans during a 9-month period from April 16, 2014, to January 15, 2015, in north central Florida. A convenience sample was recruited at various community events, and individuals were provided a $5 gift card for participation. This study was approved by the University of Florida institutional review board.

The questionnaire is described elsewhere (19,20). Questions were asked about sociodemographic characteristics (sex, age, marital status [married or not married], birthplace [in United States or not], education, employment status [employed or not], and home ownership [yes or no]), ownership and use of digital devices, health information–seeking behavior, willingness to participate in mHealth research, weight, and health status. Participants rated their overall health status on a scale from 1 to 5 (1 = excellent and 5 = poor). In addition, the questionnaire asked about sources of health information. EHL was assessed by using the eHealth Literacy Scale (eHEALS) (7,21), which is the most commonly used validated measure of EHL; eHEALS was validated with various population groups and translated into multiple languages (7,22–24). Each of the scale’s 8 items are rated on a 5-point Likert scale (1 = strongly disagree and 5 = strongly agree), and the overall score ranges from 8 to 40, with higher scores indicating greater perceived skills at finding, evaluating, and applying eHealth information to make health decisions. The internal consistency of the scale with our study sample was 0.96.

Of the 903 participants who completed the questionnaire, 881 (98%) completed all 8 eHEALS items. These participants made up the final sample. Data were analyzed using JMP PRO version 12 (SAS Institute, Inc). Descriptive data (mean, standard deviation [SD], range, median, and interquartile range) were calculated to show the dispersion of the scores (25). Cutpoints have not been validated for the eHEALS, and scores cannot be categorized reliably (7). We used the Pearson χ^2^ test, independent samples *t* tests, and one-way analysis of variance (ANOVA) to analyze data. Posthoc comparisons of ANOVA were conducted by using the Tukey–Kramer honest significant difference test. Significance was established at *P* ≤ .05 for all tests.

## Results

The mean age of the study sample was 37.0 years (SD, 14.7 y), and ages ranged from 18 to 70 years. Of 881 participants, 579 (66%) were female (Table 1). Overall eHEALS scores ranged from 8 to 40, with a mean of 30.4 (SD, 7.8), median of 32, and interquartile range of 27 to 36. The mean scores for the 8 items ranged from 3.6 (SD, 1.2) to 4.0 (SD, 1.1). The highest mean score (4.0) was for the items “I know how to find helpful health resources on the Internet” and “I know how to use the Internet to answer my health questions.” The lowest mean score (3.6) was for the items “I can tell high quality from low quality health resources on the Internet” and “I feel confident using information from the Internet to make health decisions.” (Table 2). With a total mean score of 30.8 (SD, 7.7), women had significantly higher scores than men (mean, 29.4; SD, 7.8; *t*_877_ = 7.00, *P* = .008). Scores also varied significantly by age group, education level, and employment status (*P* < .001 for all) (Table 1).

**Table 1:** Mean Scores for eHealth Literacy Scale^a^ Among African American Study Participants (N = 881), by Sociodemographic Characteristic, Florida, 2014–2015

Characteristic	No. (%)	Score, Mean (SD)	*t* Statistic or *F* Statistic (*P* Value)
**Sex** b			
Male	300 (34)	29.4 (7.8)	7.00 (.008)
Female	579 (66)	30.8 (7.7)	
**Age group^c^**			
18–29	357 (41)	31.4 (6.6)^d^	28.06 (<.001)
30–50	316 (36)	31.4 (7.2)^d^	
≥51	204 (23)	26.9 (9.4)^e^	
**Marital status** b			
Married	283 (32)	29.6 (8.6)	1.92 (.06)
Not married	594 (68)	30.72 (7.4)	
**Born in the United States** b			
Yes	783 (89)	30.2 (7.8)	1.62 (.11)
No	96 (11)	31.6 (7.8)	
**Education^c^**			
<High school	76 (9)	25.0 (10.4)^f^	20.25 (<.001)
High school	221 (25)	28.4 (8.8)^e^	
College credits	329 (38)	31.4 (6.4)^d^	
4-year degree	129 (15)	32.6 (6.0)^d^	
Graduate degree	121 (14)	32.4 (6.8)^d^	
**Employment** b			
Employed	548 (62)	31.4 (6.8)	5.31 (<.001)
Unemployed	330 (38)	28.6 (8.9)	
**Home ownership** b			
Yes	255 (29)	30.5 (7.8)	0.15 (.88)
No	624 (71)	30.4 (7.7)	

Abbreviation: SD, standard deviation.

a Each of the 8 items were rated on a 5-point Likert scale, with 1 = strongly disagree and 5 = strongly agree. Overall eHealth Literacy Scale score ranges from 8 to 40.

b *t *test performed.

c *F* test performed.

d,e,f Posthoc comparisons were conducted to determine significant differences between categories; categories that do not have matching superscripted letters are significantly different.

**Table 2:** Mean Scores for Survey Items in eHealth Literacy Scale^a^, African American Study Participants (N = 881), Florida, 2014–2015

Survey Item	Mean Score (Standard Deviation)
I know how to find helpful health resources on the Internet.	4.0 (1.1)
I know how to use the Internet to answer my health questions.	4.0 (1.1)
I know what health resources are available on the Internet.	3.7 (1.1)
I know where to find helpful health resources on the Internet.	3.8 (1.1)
I know how to use the health information I find on the Internet to help me.	3.9 (1.1)
I have the skills I need to evaluate the health resources I find on the Internet.	3.7 (1.0)
I can tell high quality from low quality health resources on the Internet.	3.6 (1.2)
I feel confident using information from the Internet to make health decisions.	3.6 (1.1)
Mean overall score	30.4 (7.8)

a Each of the 8 items were rated on a 5-point Likert scale, with 1 = strongly disagree and 5 = strongly agree. Overall eHealth Literacy Scale score ranges from 8 to 40.

Most participants owned smartphones (71%) or laptops (69%). eHEALS scores were higher among device owners than among nonowners (*P* < .01 for both). Most (70%) participants believed the Internet is useful for making health decisions. The Internet was accessed primarily from smartphones (73%) and from computers at home (71%) and at work or school (56%). eHEALS scores were significantly higher among those who accessed the Internet from smartphones and from computers at home and at work or school than among those who did not (*P* < .01 for all).

Health was rated as excellent by 15% of study participants, very good by 34%, good by 35%, fair by 15%, and poor by 2%. eHEALS scores varied significantly by self-rated health status (*F*_4,872_ = 11.49, *P* < .001). In posthoc comparisons, those who rated their health as excellent or very good had significantly higher scores than those who rated their health as good, fair, or poor. Those who rated their health as very good or good had significantly higher scores than those who rated their health as fair or poor.

Self-Rated Health StatuseHEALS Score, Mean (SD)Excellent31.4 (8.6)Very good32.1 (6.8)Good29.8 (7.8)Fair27.3 (7.8)Poor26.0 (8.8)

No other significant differences were found. Most (76%) participants reported having had a physical examination by a physician within the previous 12 months: these participants had significantly higher eHEALS scores than those who had not had an examination (mean, 31.0; SD, 7.7 vs mean, 28.4; SD, 7.7; *t*_879 _= 17.47, *P* < .001).

Health information was obtained from various sources: 62% of participants obtained health information from physicians, 60% from the Internet, and 40% from television (Table 3). eHEALS scores were significantly higher among those who cited the Internet as a source of health information than among those who did not cite this source, among those who cited nurses than among those who did not cite this source, and those who cited books, radio, or news apps as sources of information than among those who did not cite those sources.

**Table 3:** Mean Scores for eHealth Literacy Scale^a^ Among African American Study Participants (N = 881), by Source of Health Information Used, Florida, 2014–2015

Source of Health Information	No. (%)	Score, Mean (SD)	*t*_879_ (*P* Value)
**Physicians**			
Yes	542 (62)	30.5 (7.5)	0.59 (.44)
No	339 (38)	30.1 (8.2)	
**Internet**			
Yes	529 (60)	32.3 (6.1)	85.97 (<.001)
No	352 (40)	27.5 (9.0)	
**Television**			
Yes	353 (40)	30.1 (7.6)	0.80 (.37)
No	528 (60)	30.6 (7.9)	
**Nurses**			
Yes	324 (37)	31.1 (7.3)	4.53 (.03)
No	557 (63)	29.9 (8.0)	
**Books**			
Yes	290 (33)	31.3 (7.0)	6.09 (.01)
No	591 (67)	29.9 (8.1)	
**Friends**			
Yes	262 (30)	30.7 (6.6)	0.83 (.36)
No	619 (70)	30.2 (8.2)	
**Magazines**			
Yes	199 (23)	31.0 (6.9)	1.58 (.21)
No	682 (77)	30.2 (8.0)	
**Newspapers**			
Yes	153 (17)	31.0 (7.2)	1.17 (.28)
No	728 (83)	30.2 (7.9)	
**Radio**			
Yes	126 (14)	31.9 (7.0)	5.42 (.02)
No	755 (86)	30.1 (7.9)	
**News apps on smartphones**			
Yes	113 (13)	32.3 (6.2)	7.76 (.006)
No	768 (87)	30.1 (8.0)	
**Spouse or partner**			
Yes	87 (10)	31.0 (7.5)	0.68 (.41)
No	794 (90)	30.3 (7.8)	

Abbreviation: SD, standard deviation.

a Each of the 8 items in eHealth Literacy Scale were rated on a 5-point Likert scale, with 1 = strongly disagree and 5 = strongly agree. Overall eHealth Literacy Scale score ranges from 8 to 40.

**Figure 1:**
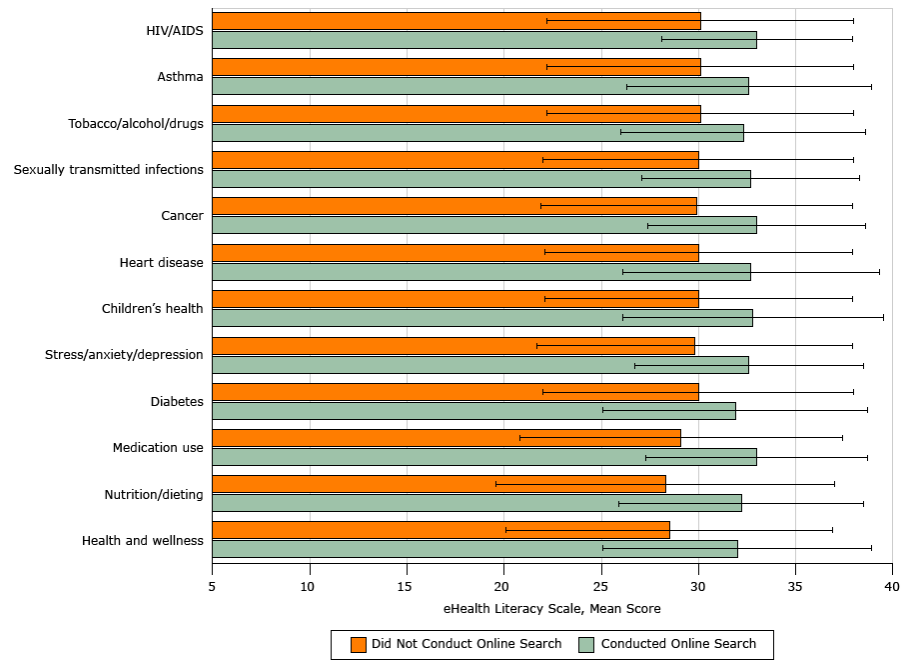
Comparison of mean eHealth Literacy Scale scores for participants who did conduct or did not conduct online searches for information in the previous 12 months, Florida, 2014–2015. A convenience sample of 881 African American adults in north central Florida were surveyed. Overall scores for the 8-item scale range from 8 to 40. All differences were significant (*P* < .01 for all). Error bars indicate standard deviation. Abbreviations: HIV/AIDS, human immunodeficiency virus/AIDS. **Table:** 

In the previous 12 months, participants reported Internet searches on the following topics: health and wellness (54% of participants), nutrition/dieting (53%), medication use (32%), diabetes (21%), stress/anxiety/depression (19%), children’s health (15%), heart disease (14%), cancer (14%), sexually transmitted infections (12%), tobacco/alcohol/drugs (12%), asthma (9%), and human immunodeficiency virus/AIDS (8%). Participants who searched online for these topics had significantly higher eHEALS scores than those who did not (*P* < .01 for all) (Figure 1).

Health Topic	Conducted Online Search, Mean Score (SD)	Did Not Conduct Online Search, Mean Score (SD)
Health and wellness	32.0 (6.9)	28.5 (8.4)
Nutrition/dieting	32.2 (6.3)	28.3 (8.7)
Medication use	33.0 (5.7)	29.1 (8.3)
Diabetes	31.9 (6.8)	30.0 (8.0)
Stress/anxiety/depression	32.6 (5.9)	29.8 (8.1)
Children’s health	32.8 (6.7)	30.0 (7.9)
Heart disease	32.7 (6.6)	30.0 (7.9)
Cancer	33.0 (5.6)	29.9 (8.0)
Sexually transmitted infections	32.7 (5.6)	30.0 (8.0)
Tobacco/alcohol/drugs	32.3 (6.3)	30.1 (7.9)
Asthma	32.6 (6.3)	30.1 (7.9)
HIV/AIDS	33.0 (4.9)	30.1 (7.9)

Most (67%) participants reported they would be willing to participate in mHealth research interventions that send educational text messages. Those who were willing to participate in mHealth research had significantly higher eHEALS scores than those who were not willing (*P* < .001). Women were significantly more willing to participate in mHealth research than men (odds ratio [OR], 1.4; 95% confidence interval [CI], 1.05–1.89; *P* = .02).

**Figure 2:**
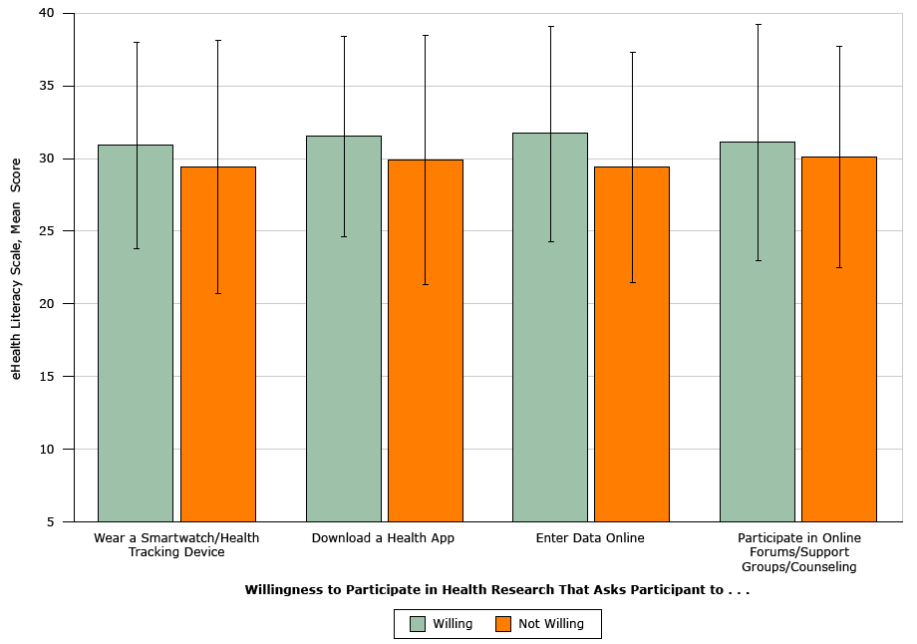
Comparison of mean eHealth Literacy Scale scores for participants willing or not willing to participate in health research that uses apps or tools, Florida, 2014–2015. A convenience sample of 881 African American adults in north central Florida were surveyed. Overall scores for the 8-item scale range from 8 to 40. Error bars indicate standard deviation. **Table:** 

Many (41%) participants reported having downloaded a nutrition/health/fitness app in the previous 30 days. Those who had downloaded an app had significantly higher eHEALS scores than those who had not (mean score, 32.6 [SD, 6.3] vs mean score, 28.9 [SD, 8.2]; *t*_879 _= 50.73, *P* < .001). Women were significantly more likely than men to have downloaded an app (OR, 1.61; 95% CI, 1.21–2.16; *P* = .001). Participants reported willingness to participate in research that used smartwatches/health tracking devices (62% of participants), health apps (57%), online data entry (42%), or online forums/support groups/counseling (29%). Women were significantly more likely than men to report they would be willing to participate in research that asked them to wear a smartwatch/health tracking device (OR, 1.45; 95% CI, 1.09–1.93; *P* = .01), enter data online (OR, 1.75; 95% CI, 1.31–2.33; *P* < .001), or participate in online forums/support groups/counseling (OR, 1.69, 95%; CI, 1.22–2.33; *P* = .002). eHEALS scores were significantly different between those who were willing to participate in research that asked them to wear a smartwatch/health tracking device (*t*_879_ = 7.71; *P* = .006), download a health app (*t*_879 _= 24.29; *P* < .001), or enter data online (*t*_879 _=18.06; *P* < .001) compared with those who were not willing. We found no significant differences in eHEALS scores for those willing to participate in research that used online forums/support groups/counseling and those not willing (Figure 2).

Willingness to Participate in Health Research That Asks Participant to . . .	Willing, Mean Score (SD)	Not Willing, Mean Score (SD)
Wear a smartwatch/health tracking device	30.9 (7.1)	29.5 (8.7)
Download a health app	31.5 (6.9)	29.9 (8.6)
Enter data online	31.7 (7.4)	29.4 (7.9)
Participate in online forums/support groups/counseling	31.1 (8.1)	30.1 (7.6)

## Discussion

Understanding the association between EHL, access to technology, health information–seeking behavior, and willingness to participate in mHealth research is an important step in creating mHealth messages, programs, and interventions to prevent and manage chronic diseases among African Americans. The percentage of study participants who owned a smartphone (71%) was higher than the percentage of the general population (68%) who own one (26). Study participants also used smartphones as their primary Internet access, which allows them to check signs or symptoms of health conditions, find free information on nutrition, and self-monitor weight, physical activity, and other health-related factors (1,27).

Most participants had eHEALS scores above the mean. However, the study found low scores on 2 items: the ability to differentiate high-quality from low-quality health resources on the Internet and confidence in using information from the Internet to make health decisions. These low scores are a key finding because participants reported searching online for information on many health topics. The inability to differentiate between credible and noncredible online health information and sites can make patients vulnerable to exploitation (28,29). Furthermore, low confidence in using online information to make health decisions suggests that credible Web portals and apps need to develop algorithms to help personalize information for users with various education and literacy levels (30,31).

Physicians and the Internet were the primary sources of health information used by participants with no significant differences in eHEALS scores, but surprisingly those who reported nurses as a source of health information had significantly higher eHEALS scores than those who did not. Nurses may be perceived as having more time to answer questions and as having better interpersonal communication skills than physicians (28,32). Because patients often use online information to manage their health condition (33), practitioners should consider providing patients with a list of credible sites that can answer questions and concerns. Such a list could also enhance patients’ confidence and improve face-to-face interaction with practitioners (34).

As expected, eHEALS scores varied by educational level. Although people with low income and low educational levels are less likely to use the Internet than people with high income and high educational levels, they are more likely to search for health information than any other topic when they do use the Internet (35). Women also had significantly higher eHEALS scores than men and expressed a greater willingness to participate in mHealth research in general and to participate in research that asked them to download health apps or wear tracking devices. These findings are consistent with the findings of other studies that show African American women express greater interest in health issues than African American men (36). Thus, as gatekeepers to the home and a major source of informal health information for the men in their lives, African American women should be considered as primary and secondary targets for mHealth interventions for various health conditions (19).

This study has several limitations. First, it used a convenience sample, which limits the generalizability of the findings to African Americans in north central Florida. Second, survey data are subject to social desirability bias. Third, only a few instruments exist to measure EHL, and a different survey instrument could have given different results. eHEALS is not a clinical diagnostic tool, and scores should be interpreted cautiously because appropriate cutpoints have not been validated (7). However, the scale is the most common measure of EHL, and it was validated among various population groups and translated into multiple languages (7,22–24). Furthermore, eHEALS has the potential to identify people who may benefit from using online resources and screening people who may benefit from mHealth interventions (7).

We found higher eHEALS scores among women, smartphone owners, those who used the Internet as a source of health information, and those willing to participate in research interventions that send educational text messages. These findings may be beneficial to practitioners, researchers, and program planners as they explore new strategies for developing, tailoring, and delivering online health information and interventions to prevent chronic diseases among African Americans. Further research is needed to develop new EHL assessment tools or improve existing ones for use in clinical settings and mHealth interventions. Additionally, research is needed in developing, implementing, and evaluating the content, format, and usefulness of online health information for low-income African American and those with low levels of literacy.
